# Pulmonary Nodules After Leflunomide Therapy in Rheumatoid Arthritis: A Case Report

**DOI:** 10.1002/ccr3.9615

**Published:** 2024-12-30

**Authors:** Mahdiye Abiyarghamsari, Arman Ahmadzadeh

**Affiliations:** ^1^ Department of Clinical Pharmacy, School of Pharmacy Shahid Beheshti University of Medical Sciences Tehran Iran; ^2^ Department of Internal Medicines Loghman Hakim Hospital, Shahid Beheshti University of Medical Sciences Tehran Iran; ^3^ Department of Rheumatology Loghman Hakim Hospital, Shahid Beheshti University of Medical Sciences Tehran Iran

**Keywords:** leflunomide, nodules, pulmonary, rheumatoid arthritis

## Abstract

Rheumatoid arthritis (RA) is a systemic autoimmune disorder with both articular and extra‐articular manifestations, including rare pulmonary complications. We report a case of a 65‐year‐old male with long‐standing RA who developed multiple cavitary pulmonary nodules following prolonged leflunomide therapy. Diagnostic evaluation excluded infectious, neoplastic, and autoimmune causes. Bronchoscopy and biopsy confirmed an acute suppurative inflammatory process with central necrosis. Discontinuation of leflunomide resulted in the resolution of pulmonary nodules. This case highlights the need for vigilance regarding potential pulmonary toxicity in RA patients receiving leflunomide and underscores the importance of prompt recognition and management of drug‐induced complications.

**Summary**

- This is a case of a rheumatoid arthritis patient who developed symptomatic necrobiotic lung lesions after treatment with leflunomide.

## 1 Introduction

Rheumatoid arthritis (RA) is a systemic inflammatory disorder characterized by synovitis, leading to joint destruction and affecting various extra‐articular systems like the mucocutaneous, cardiovascular, pulmonary and renal systems [1, 2]. Peripheral rheumatoid nodules are a prevalent and highly specific dermatological manifestation, whereas pulmonary nodules in RA occur less frequently [3, 4]. Although pulmonary nodules represent a known complication of RA, a dedicated therapeutic strategy is lacking. Risk factors for pulmonary nodules include male sex, longer disease duration, presence of subcutaneous rheumatoid nodules, rheumatoid factor (RF) positivity, and smoking [5]. Notably, these nodules can also develop as an adverse effect of RA medications, particularly methotrexate. This highlights the need for careful vigilance in managing RA patients, considering both the intrinsic risk of lung involvement and potential drug‐related complications [6, 7]. Leflunomide is a common DMARD that inhibits dihydroorotate dehydrogenase and tyrosine kinase to downregulate T and B lymphocyte proliferation [8]. While its efficacy in controlling joint inflammation in RA patients is well‐established, emerging evidence has shed light on a potential association between leflunomide use and the development of pulmonary nodules [9]. The putative link between leflunomide and the induction of pulmonary nodules raises significant concerns, necessitating a meticulous exploration of the underlying mechanisms and clinical implications.

## 2 Case History/Examination

The 65‐year‐old male patient, previously diagnosed with rheumatoid arthritis (RA) for the past 20 years, sought evaluation at the emergency department on November 14, 2023, citing dyspnea as the chief complaint. Notably, the patient had been hospitalized the previous month due to dyspnea and decreased arterial oxygen saturation, which prompted further investigative work‐up. The onset of dyspnea was acute, devoid of any antecedent symptoms, and notably intensified during exertional activities surpassing his customary level.

The patient does not report a history of cough, phlegm, fever, chills, or chest pain. Clinically, the patient is a middle‐aged man with a height of 185 cm and a weight of 82 kg, who appears non‐toxic and ill. He does not mention any history of weight loss or nocturnal sweats. However, he suffers from reflux disease.

In the musculoskeletal examination, severe inflammation in the hand joints and pronounced ulnar deviation were evident, and the patient complained of pain in the interphalangeal joints.

The patient reports a history of smoking, approximately one to two cigarettes per day. The patient's medication history includes Tab Valsartan/Amlodipine 80/5 QD, Tab Atorvastatin 40 QD, Tab Quetiapine 100 1/2 QD, Tab Citalopram 20 QD, Tab Omeprazole 20 BD, Tab Leflunomide 20 QD, Tab Prednisolone 5 QD, Tab Folic Acid 5 BD.

The patient had a history of using sulfasalazine, which was switched to methotrexate due to lack of efficacy. However, the patient was unable to tolerate oral methotrexate, so the treatment was changed to an injectable form. This, too, proved intolerable, leading to a further switch to leflunomide.

## 3 Methods

At the initial physical examination, the patient's blood pressure was recorded at 115/85 mmHg, heart rate at 82 beats per minute, respiratory rate at 16 breaths per minute, and temperature at 36.7°C. The patient does not appear pale, and no signs of cyanosis were observed. The results of the ancillary tests were as Table 1. Also, angiotensin converting enzyme (ACE) level was not elevated, which was performed to exclude sarcoidosis.

**TABLE 1:** Laboratory results.

Lab test	Result	Normal range	Lab test	Result	Normal range
WBC	9.6	4–11.5 × 10^3^/μL	Ca	9.5	8.5–10.5 mg/dL
RBC	4.41	4–6 × 10^6^/μL	Na	139.2	134–147 mEq/L
Hb	13.1	13.5–17.5 g/dL	**K**	5.37	3.5–5.1 mEq/L
HCT	40.3	41.5%_50.5%	Urea	40	15–45 mg/dL
MCV	91.4	80–100 fL	Cr	1.2	0.6–1.5 mg/dL
MCH	29.7	26–34 pg	**RF**	59	0–20 IU/mL
MCHC	32.5	31–37 g/dL	**ESR**	62	Up to 20 mm/h
PLT	357	150–450 × 103/μL	**CRP**	39	0–6 mg/L
RDW	11	10%–17.9%	**25 (OH) Vitamin D3**	27	30–100 ng/mL

Abbreviations: Ca, calcium; Cr, creatinine; CRP, C‐reactive protein; ESR, erythrocyte sedimentation rate; Hb, hemoglobin; HCT, hematocrit; K, potassium; MCH, mean corpuscular hemoglobin; MCHC, mean corpuscular hemoglobin concentration; MCV, mean corpuscular volume; Na, sodium; PLT, platelet count; RBC, red blood cell; RDW, red blood cell distribution width; RF, rheumatoid factor; WBC, white blood cell.

*Note:* Bold values indicate results outside the normal reference range and are considered clinically significant.

**FIGURE 1:**
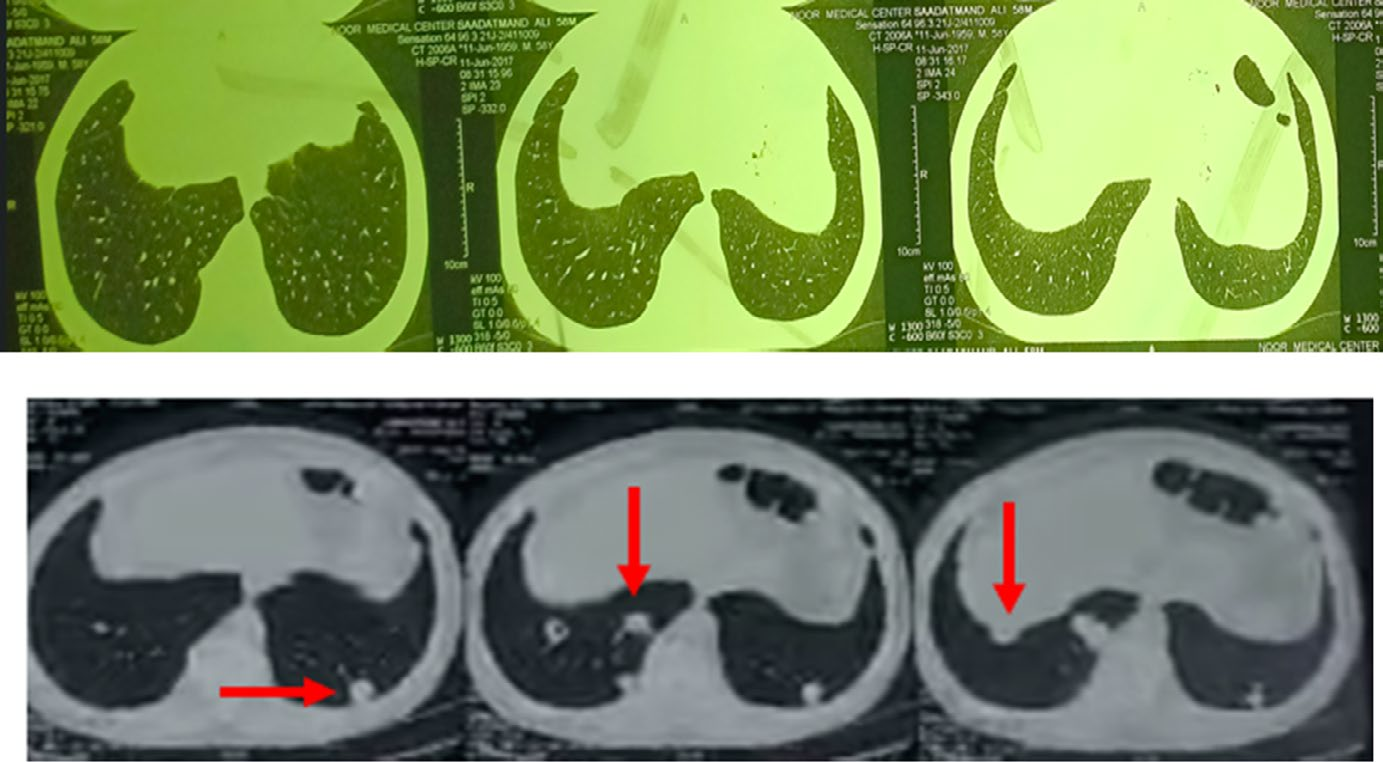
Chest CT axial images, Upper panel, before leflunomide therapy. Lower panel, after leflunomide therapy, showing multiple rheumatoid nodules.

A Computed Tomography (CT) scan of the chest was performed at the onset of dyspnea, revealing no evidence of pulmonary embolism but showing multiple cavitary pulmonary nodules, predominantly in the left lung base (Figure 1).

Viral, bacterial, and parasitic antibodies were within normal limits. Tuberculin skin test and Interferon Gamma Release Assay (IGRA) test were negative.

Microscopic and macroscopic analysis of the patient's urine sample was conducted, yielding no significant findings (Table 2).

**TABLE 2:** Urine analysis.

Macroscopic		Microscopic	
Color	Yellow	WBC	0–2
Appearance	Clear	RBC	1–2
Specific Gravity	1.015	Epithelial Cells	0–2
pH	5	Bacteria	Negative
Protein	Negative	Mucus	Negative
Glucose	Negative	Casts	Negative
Bilirubin	Negative	**Crystals**	Ca‐Oxalate: 5–10
Urobilinogen	Normal		
Ketone	Negative		
Nitrite	Negative		
Blood	Negative		

Abbreviations: RBC, red blood cell; WBC, white blood cell.

*Note:* Bold values indicate results outside the normal reference range and are considered clinically significant.

Bronchoscopy result was normal; Bronchoalveolar Lavage (BAL) was negative for Acid‐Fast Bacillus (AFB), BAL cultures for mycobacteria and fungi were negative, and BAL cytology revealed no neoplastic cells. A biopsy of a peripheral nodule revealed an acute suppurative inflammatory process with central necrosis. In view of this result, which ruled out neoplastic and infectious disease, it was decided to discontinue leflunomide. At the time, it was decided that the patient should undergo clinical and radiological follow‐up. After a few months following the discontinuation of leflunomide, the pulmonary nodules disappeared.

## 4 Conclusion and Result

The prevalence of RA nodules exhibits variability, with detection rates varying across diagnostic modalities. Notably, their detection through open lung biopsy reaches as high as 32%.

The manifestation of pulmonary nodules in RA patients poses a diagnostic challenge, given the diverse differential diagnoses associated with similar presentations. Conditions such as malignancy, bacterial and fungal infections, vasculitis, and tuberculosis can mimic the presentation of RA nodules; however, the absence of systemic symptoms is uncommon. Nodules due to lung cancer is characteristically greater than 10 mm in diameter and have irregular borders. Metastases can also appear as multiple nodules in the lungs.

Consequently, a comprehensive differential diagnosis must encompass the aforementioned conditions. In our investigation, we thoroughly evaluated the differential diagnosis possibilities for our case and discerned the absence of any pathological findings. Furthermore, the biopsy results corroborated the diagnosis of rheumatoid pulmonary nodule.

While there are numerous ambiguities surrounding this adverse effect of leflunomide, including its mechanism, onset, risk factors, and course of improvement and treatment exist, discontinuation of leflunomide remains the primary intervention highlighted across various primary articles. In some patients, cholestyramine has been prescribed to hasten drug elimination due to its long half‐life, while steroid therapy has been utilized as adjunctive treatment in certain cases. Additionally, patients have received another immunosuppressant to manage their underlying condition. These patients require close monitoring, with repeat CT scans to assess the status of nodules. However, the precise timing for this follow‐up and resolution of nodules post‐leflunomide discontinuation remains undefined.

In summary, medication‐associated nodulosis should be recognized as a rare complication in rheumatoid arthritis patients treated with leflunomide.

## 5 Discussion

The term ‘accelerated nodulosis’ describes the swift advancement and/or spread of new or existing nodules. These nodules, while clinically and histologically identical to typical rheumatoid nodules, exhibit distinctive characteristics such as rapid onset and growth, occurrence despite low disease activity, smaller size, and a preference for the fingers, feet, and ears [10]. The first description of accelerated nodules was in 1986 by Kremer and Lee, who reported their occurrence in three out of 29 RA patients treated with methotrexate (MTX) [11]. Accelerated nodulosis has also been reported in association with other drugs, such as tumor necrosis factor (TNF alpha) inhibitors, azathioprine, leflunomide, aromatase inhibitors, and tocilizumab [12, 13, 14, 15]. Therefore, some have proposed the terminology “immunomodulatory agents induced nodulosis”. The attributed causality between accelerated nodules and the immunomodulatory drugs is due to the observation that nodules usually regress after drug withdrawal and may recur upon drug rechallenge.

There are several mechanisms, different for each medication, proposed to potentate accelerated development of rheumatoid nodules [16]. However, this manifestation has never been observed in cancer patients treated with methotrexate, suggesting that the pathogenetic mechanisms of inflammatory diseases are of special importance [10]. A proposed mechanism of leflunomide associated nodulosis is reduction in monocyte activity leading to reduced elimination of RF. In turn, RF accumulation in reticuloendothelial system alveolar macrophages becomes a nidus for rheumatoid nodule formation. The majority of cases are asymptomatic or exhibit few symptoms. Reported symptoms include cough, dyspnea, and chest pain [9].

Although clinical reports of pulmonary nodules exist (Table 3), the literature concerning leflunomide‐induced nodules remains limited. Thus, there is a pressing need for a comprehensive and evidence‐based examination of existing case studies, clinical trials, and experimental research.

**TABLE 3:** The reports of pulmonary nodules following the use of leflunomide.

References	Age (year)	Gender	Duration of disease (year)	Leflunomide duration (months)	Rheumatoid factor	Smoking	Presence of subcutaneous nodule
Yoshikawa et al. [6]	60	F	10	NM	+	NM	NM
Horvath et al. [16]	34	F	9	36	+	−	−
Kim et al. [17]	46	F	2	20	+	−	−
Rozin et al. [18]	77	M	18	13	+	+	+
Rozin et al. [18]	66	M	22	7	+	+	+
Balkarli et al. [8]	60	F	5	20	+	+	−
Balkarli et al. [8]	42	F	20	13	+	−	−
Balkarli et al. [8]	42	F	9	15	+	−	−
Balkarli et al. [8]	65	M	14	10	+	−	−
Springsted et al. [19]	49	F	NM	36	+	NM	NM

Abbreviations: F, female; M, male; NM, not mentioned.

Understanding the specific characteristics, prevalence, and potential risk factors associated with leflunomide‐induced pulmonary nodules is essential for healthcare practitioners prescribing this DMARD and for patients undergoing long‐term treatment.

## Author Contributions

**Mahdiye Abiyarghamsari:** writing – original draft. **Arman Ahmadzadeh:** project administration, supervision, validation.

## Consent

Informed consent was obtained from the patient at the time of development of this manuscript. Written consent has been obtained.

## Conflicts of Interest

The authors declare no conflicts of interest.

## Data Availability

The data that support the findings of this study are available from the corresponding author upon reasonable request.
